# Donor Dependent Variations in Hematopoietic Differentiation among Embryonic and Induced Pluripotent Stem Cell Lines

**DOI:** 10.1371/journal.pone.0149291

**Published:** 2016-03-03

**Authors:** Olivier Féraud, Yannick Valogne, Michael W. Melkus, Yanyan Zhang, Noufissa Oudrhiri, Rima Haddad, Aurélie Daury, Corinne Rocher, Aniya Larbi, Philippe Duquesnoy, Dominique Divers, Emilie Gobbo, Philippe Brunet de la Grange, Fawzia Louache, Annelise Bennaceur-Griscelli, Maria Teresa Mitjavila-Garcia

**Affiliations:** 1 INSERM UMR935, SFR André Lwoff, University Paris Sud, Paul Brousse Hospital, Villejuif, France; 2 Human Embryonic Stem Cell Core Facility, ESTeam Paris Sud, Villejuif, France; 3 Department of Pediatrics and Center for Tropical Medicine and Infectious Diseases, Texas Tech University Health Science Center, Amarillo, TX, United States of America; 4 INSERM UMR U1170, Institut Gustave Roussy, Villejuif, France; 5 AP-HP, Department of Hematology, University Hospitals Paris Sud, Paul Brousse Hospital, Villejuif, France; 6 Faculty of Medicine, Kremlin-Bicêtre, University, Paris Sud, Paris Saclay, France; 7 Sanofi, Department of Regenerative Medicine, Global Biotherapeutics, Vitry-sur-Seine, France; 8 INSERM/UMR S933, University Pierre and Marie Curie-Paris 6, Paris, France; 9 INSERM UMR 972, SFR André Lwoff, Paul Brousse, Hospital, Villejuif, University Paris Sud, Paris Saclay, France; 10 National Infrastructure INGESTEM, University Paris Sud, Inserm, Paris, France; University of Minnesota, UNITED STATES

## Abstract

Hematopoiesis generated from human embryonic stem cells (ES) and induced pluripotent stem cells (iPS) are unprecedented resources for cell therapy. We compared hematopoietic differentiation potentials from ES and iPS cell lines originated from various donors and derived them using integrative and non-integrative vectors. Significant differences in differentiation toward hematopoietic lineage were observed among ES and iPS. The ability of engraftment of iPS or ES-derived cells in NOG mice varied among the lines with low levels of chimerism. iPS generated from ES cell-derived mesenchymal stem cells (MSC) reproduce a similar hematopoietic outcome compared to their parental ES cell line. We were not able to identify any specific hematopoietic transcription factors that allow to distinguish between good *versus* poor hematopoiesis in undifferentiated ES or iPS cell lines. There is a relatively unpredictable variation in hematopoietic differentiation between ES and iPS cell lines that could not be predicted based on phenotype or gene expression of the undifferentiated cells. These results demonstrate the influence of genetic background in variation of hematopoietic potential rather than the reprogramming process.

## Introduction

Human embryonic stem cells (ES) isolated from the inner cell mass of a blastocyst and human induced pluripotent stem cells (iPS) lines derived from fetal or adult tissues, have the ability to self-renew indefinitely while maintaining their pluripotency to differentiate into multiple cell lineages [1–3]. ES and iPS cells are able to differentiate into all hematopoietic lineages [4–8], however identification of a multipotent engraftable hematopoietic stem cell remains a challenge. Generation of *ex-vivo* multipotent hematopoietic stem cells from ES and iPS cells may serve as an alternative source for long-term *in vivo* hematopoietic reconstitution and for understanding early stages of hematopoietic development in normal and pathological contexts.

Many ES cell lines have been characterized for their hematopoietic potential in different studies but only few iPS cell lines have been characterized in detail [3,5,7]. Lineage-specific differentiation potential varies among different pluripotent stem cells (PSC) [5,9–12] however variations in hematopoietic differentiation among iPS cell lines have not been widely addressed. In the current study, we used improved hematopoietic differentiation protocols to compare the hematopoietic potential of 4 ES and 14 iPS cell lines of various origins. We found significant intrinsic variations in hematopoietic differentiation ability *in vitro* in both ES and iPS cell lines from different individuals. Reprogramming of ES-derived MSC did not modify this intrinsic hematopoietic potential and isogenic iPS-derived MSC-ES reproduces a similar hematopoietic outcome as their parental ES cell line. In addition, we investigated whether the variation in hematopoietic differentiation among different ES and iPS cell lines could be predicted by expression of key genes involved in hematopoiesis. A large variation in the level of gene expression at the pluripotent stage was observed but was not able to be correlated to distinguish PSC lines with greater hematopoietic potential. As expected, the expression level of these key hematopoietic factors varied during hematopoietic differentiation. The ability of ES and iPS-derived MSC-ES cell lines to allow *in vivo* hematopoietic reconstitution in immunodeficient mice was detected at low levels for short-term engraftment. Our results show that there is an intrinsic variability of each cell line regarding the hematopoietic differentiation potential. It appeared that the donor cell of origin is a determinant factor for variations in iPS hematopoietic differentiation rather than the derivation or induction methods. These data underline the importance of cell donors for hematopoietic differentiation potential from iPS cell lines.

## Materials and Methods

### Pluripotent stem cells

ES cell lines used were SA01 from Cellartis AB, (Sweden), H1 and H9 from WiCell Research Institute (Madison, WI, USA, http://www.wicell.org) imported after authorization from Biomedicine Agency (number RE10-035R/C) and CL01, ES cell line derived by our laboratory from a PGD embryo harboring a trisomy 1 and monosomie 21 (www.hescreg.eu). In addition, fourteen iPS cell lines were studied: two iPS derived from MSC obtained from SA01 and H9 cell lines (iPS-MSC-SA01 and iPS-MSC-H9), ten iPS from healthy donors and two iPS from sickle cell diseases. CL01 human ES cell line and all iPS cell lines were derived by the StemCell core facility (ESTeam Paris Sud) with written and informed consent from the donors in accordance with the approval from the ethical committee of Department of Medicine at Kremlin Bicêtre University Paris Sud (Comité de protection des personnes "CPP Ile-de-France VII", Hôpital de Bicêtre, 78 rue du Général-Leclerc, 94270 Le Kremlin-Bicêtre, cpp.idf.7-bicetre@wanadoo.fr). Detailed information about iPS lines (PB3 to PB33) is described in Table 1. iPS cell lines (PB3 to PB33) were derived using lentiviral vectors carrying the following transgenes *OCT4*, *SOX2*, *NANOG*, *LIN28*, or *OCT4*, *SOX2* under the control of the elongation factor-1 promoter, using published methods [3], or by retroviral vectors or by Sendai virus carrying the following transgenes *OCT4*, *SOX2*, *KLF4*, *C-MYC* [1,13]. The iPS-MSC-ES cell lines (iPS-MSC-SA01 and iPS-MSC-H9) were derived using lentiviral vectors expressing *OCT4*, *SOX2*, *NANOG*, *LIN28* as we have previously described [14]. Briefly, MSC were generated by culturing SA01 and H9 ES cell lines in DMEM/F12 (Invitrogen) medium, supplemented with 10% heat-inactivated FBS (Hyclone), 1 ng/mL bFGF, 0.1mM non-essential amino acids, 1mM L-glutamine, 0.1mM β-mercaptoethanol and 1% penicillin-streptomycin. At 4 weeks adherent cells were isolated and characterized by flow cytometry for markers to MSC differentiation lineage and to negativity for pluripotent markers *OCT4*, *SOX2*, *NANOG*, and *LIN28* and tested for differentiation along the osteogenic, chondrogenic and adipogenic lineages [14]. MSC void of any pluripotency were then used to generate iPS as described above. Pluripotency of all iPS was confirmed by teratoma assay and histology of representative tissue from three germ layers, expression of pluripotent markers (TRA-1-60, TRA-1-81, SSEA-3 and SSEA-4) by flow cytometry. iPS cell lines maintaining the expression of transgene as analyzed by reverse transcription polymerase chain reaction (RT-PCR) were exclude from these study. Genomic integrity was assessed by karyotype analysis [14,15].

**Table 1:** Cell’s origin, reprogrammation method, characterization and hematopoietic analysis of iPS.

hiPS	Cell origin	Reprogrammation genes	Method	Passage (range)	n =	CFC (range)
PB3	normal amniocytes	OSLN	LV	22 to 32	3	30 to 250
PB4	amniocytes from sickle cell anemia	OSLN	LV	21 to 32	3	0 to 169
PB5	amniocytes from sickle cell anemia	OSLN	LV	25 to 28	3	0 to 0
PB7	normal amniocytes	OSKC	RV	15 to 66	6	33 to 429
PB8	normal amniocytes	OSKC	RV	19 to 28	3	0 to 0
PB9	normal amniocytes	OSLN	LV	14 to 27	3	0 to 0
PB10	normal amniocytes	OS	LV	14 to 24	3	12 to 36
PB11	normal amniocytes	OSLN	LV	15 to 71	12	103 to 2242
PB13	EPC from normal PB	OSLN	LV	18 to 44	6	27 to 832
PB17	EPC from normal PB	OSLN	LV	31 to 47	4	50 to 853
PB30	CD34+ from normal cytapheresis	OSKC	Sendai	13 to 28	3	69 to 130
PB33	CD34+ from normal BM	OSKC	Sendai	44 to 47	3	96 to 182

Abbreviations: RV: retrovirus. LV: lentivirus. OSLN: *OCT4*, *SOX2*, *LIN28*, *NANOG*; OSKC: *OCT4*, *SOX2*, *KLF4*, *C-MYC*; OS: *OCT4*, *SOX2*. EPC: Endothelial Progenitor Cells, PB: peripheral blood, BM: bone marrow. For more details: European Registry (http://www.hescreg.eu).

### Culture of ES and iPS cells

Human ES and iPS were cultured on mitomycin-treated mouse embryonic fibroblast cells in Knockout DMEM medium supplemented with 20% Knockout serum replacement (Invitrogen), 1% penicillin/streptomycin, 1 mM L-glutamine, 1% non-essential amino acids, 0.1 mM β-Mercaptoethanol (Sigma Aldrich) and 5 ng/mL basic fibroblast growth factor (bFGF, Invitrogen). Half of the medium was changed daily and cells were passaged weekly with collagenase type IV at 1 mg/mL (Invitrogen) and scrapped to obtain small clumps. ES cells were used between passage 25 and 70, iPS-MSC-ES were used between passage 20 and 65, and iPS cells were used between passage 13 and 71 (Table 1). At the same time as hematopoietic induction, pluripotency of ES and iPS cells were verified by flow cytometry with SSEA-3, SSEA-4, TRA-1-60 and TRA-1-81 (antibodies from BD Biosciences) (S1A and S1B Fig).

### ES and iPS cell differentiation toward hematopoietic lineage

**Fig 1:**
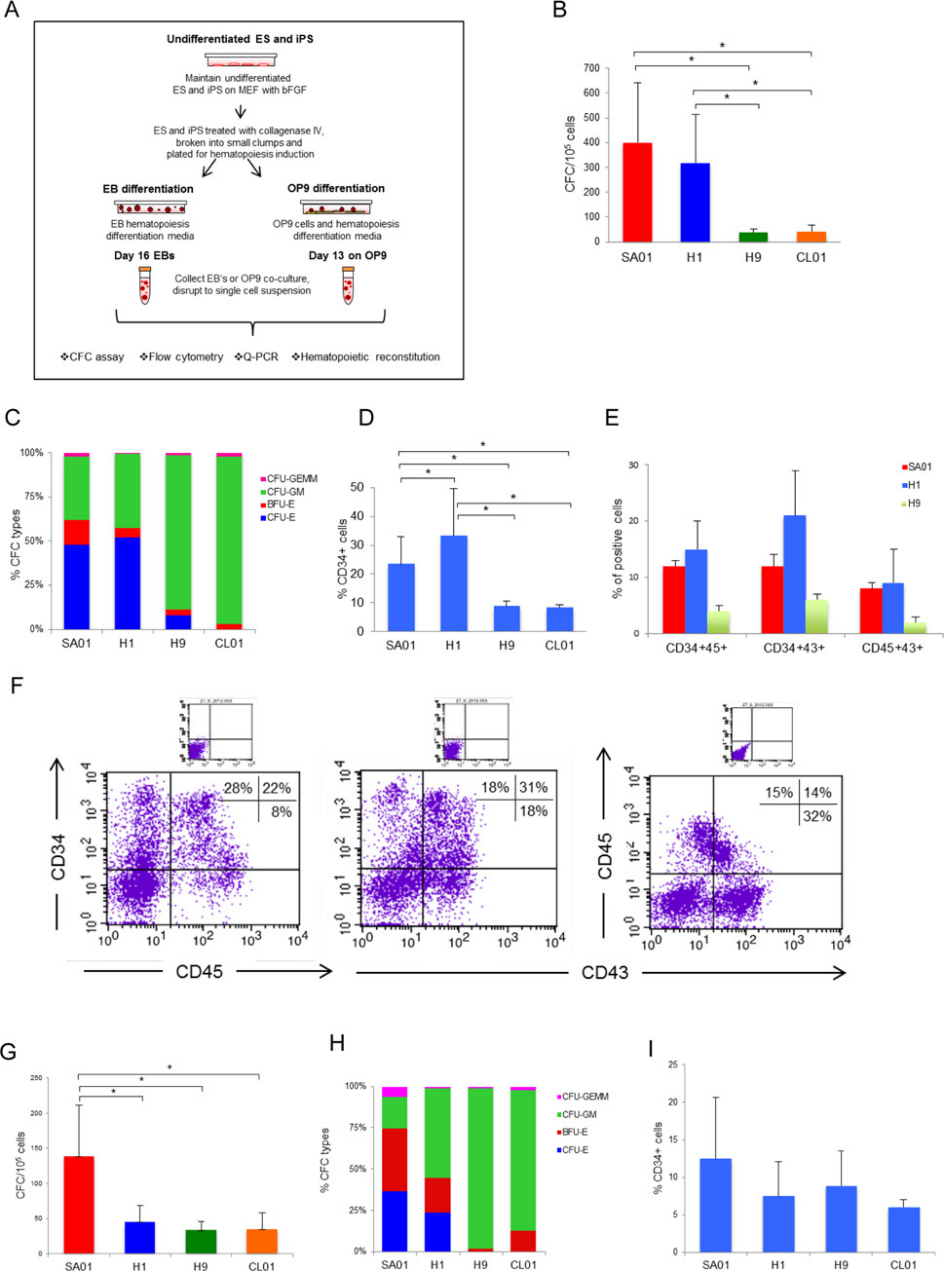
Hematopoietic differentiation potential among four ES cell lines. (A) Protocol schematic for hematopoietic differentiation by EB and OP9 co-cultures methods. ES cell lines are cultured on MEF with bFGF, treated with collagenase IV, broken into small clumps and plated for hematopoiesis induction. Clumps are seeded in EB differentiation media in ultra low attachment plates incubate at 37°C in 5% CO_2_ for 16 days, media was change 2–3 times. For OP9 co-culture, clumps are seeded in hematopoiesis differentiation media on OP9 grown to confluence and media changed at days 4, 6 and 8 cultured at 37°C in 5% CO_2_ for 13 days. At day 16 (EB) and day 13 (OP9 co-culture), cells were disrupt to single cell suspension and plate for CFC, analyze flow cytometry, Q-RT-PCR, or mouse reconstitution assay analysis. B-F) by the EB method. (B) Number of CFC counted and classified according to morphology. Each assay was performed in triplicate, data is shown as mean ± s.d. of n = 27, 34, 3 and 3 independent experiments for SA01, H1, H9 and CL01 respectively. There is no statistically significant difference for CFC number when comparing SA01 *vs* H1 and H9 *vs* CL01 (p>0.05), in contrast to SA01 *vs* H9, SA01 *vs* CL01, H1 *vs* H9, H1 *vs* CL01 are statistically significant different (*p<0.05). (C) Types of CFC (CFU-E, BFU-E, CFU-GM and CFU-GEMM). (D) Flow cytometric analysis of the percentage of CD34+ cells in EB-derived ES cells n = 12, 22, 3 and 3 for SA01, H1, H9 and CL01 respectively. Differences of expression of CD34 for SA01 *vs* H1, SA01 *vs* H9, SA01 *vs* CL01, H1 *vs* H9, H1 *vs* CL01 were statistically significant (*p<0.05). (E) EB-derived cells from SA01, H1 and H9 cell lines co-expressed CD34/CD45, CD34/CD43 and CD45/CD43 hematopoietic markers n = 4, 8 and 3, respectively. (F) Representative FACS analysis for EB-derived cells from H1 cell line co-expressed CD34/CD45, CD34/CD43 and CD45/CD43 hematopoietic markers. G-I) on OP9 co-cultures, (G) Number of CFC generated, SA01 n = 6, H1 n = 8, H9 n = 3 and CL01 n = 3, CFC number from SA01 was significantly different from that obtained from the 3 others ES cell lines (*p<0.05). (H) Distribution of CFC types. (I) Flow cytometric analysis of the percentage of CD34+ cells after OP9 differentiation, SA01 n = 6, H1 n = 8, H9 n = 7 and CL01 n = 3 were not statistically significant (p>0.05).

Formation of EB. For Embryoid Bodies (EB) formation, ES and iPS cells, at day 6–7 after cell passage, were treated with collagenase IV. Small clumps were cultured in Iscove’s modified Dulbecco’s medium (IMDM, Invitrogen) supplemented with 1% penicillin/streptomycin, 1 mM L-glutamine, 15% fetal calf serum (FCS, Invitrogen), 450 μM monothioglycerol, 50 μg/mL ascorbic acid (Sigma Aldrich) and 200 μg/L transferrin (Sigma Aldrich), as previously described [16] and supplemented with hematopoietic cytokines: 100 ng/mL stem cell factor (SCF), 100 ng/mL fms-like tyrosine kinase 3 ligand (Flt-3L) and 50 ng/mL thrombopoietin (TPO) (all from Peprotech). ES and IPS derived EB were cultured in ultra low attachment 6-well plates (Costar) for 16 days. Media was changed two or three times depending on EB proliferation. All cultures were incubated at 37°C in 5% CO_2_ (Fig 1A).

Differentiation of ES cells in co-culture with OP9 stromal cell line. For ES differentiation, we also used OP9 co-culture method described by Vodyanik [8]. Briefly, OP9 cell line (ATCC) were plated onto gelatinized dishes in α-MEM medium without ribonucleotides and deoxyribonucleotides (Invitrogen) supplemented with 1% penicillin/streptomycin, 1 mM L-glutamine, 10% FCS and 100 μM monothioglycerol (Sigma Aldrich) at 5x10^5^ cells per 100 mm dish, after 3 to 4 days cells were confluent and half of the medium was changed. Clumps of undifferentiated ES cells, at day 6–7 after ES cell passage, were added at equivalent to 1.5x10^6^ cells per dish in the same medium. At days 4, 6 and 8 half of the medium was changed and cells were further cultured for 13 days (Fig 1A).

At day 16 (EB) and day 13 (OP9 co-culture), cells were dissociated by collagenase IV (Invitrogen) treatment, and used for flow cytometry analysis, hematopoietic colony-forming cell (CFC) assays, quantitative RT-PCR (Q-RT-PCR) analysis and for *in vivo* hematopoiesis reconstitution in immunodeficient NOD/shi-SCID IL2Rγ^-/-^ (NOG) mice.

### Hematopoietic colony-forming cell assays

CFC assays from ES and iPS cells from EB or co-culture with OP9 were plated at 5x10^4^ or 10^5^ cells/mL into MethoCult GF (H4435 StemCell Technologies) in triplicate. Hematopoietic CFC (CFU-E, BFU-E, CFU-GM and CFU-GEMM) were counted at day 14 according to standard morphological criteria. It is known that the CFC assay can be highly operator-dependent, therefore we took this into account in the experimental design and the same manipulator carried out all CFC experiments.

### Flow cytometry analysis

EB were collected as previously described, the resulting single-cell suspension was incubated with the fluorochrome-conjugated antibodies (mAbs): CD34-APC, CD43-FITC and CD45-PE (antibodies from BD Biosciences).

Cells were incubated with selected antibodies for 30 minutes on ice, washed and resuspended in PBS containing 5% FCS and 7-aminoactinomycin D (7AAD; 1μg/mL) (Sigma Aldrich) to exclude dead cells. Isotype matched FITC-, PE- and APC-conjugated irrelevant mAbs (BD Biosciences and Beckman Coulter). Analysis was carried out with a FACSCalibur flow cytometer using CellQuest Pro Analysis software (Becton-Dickinson).

### Hematopoietic repopulating activity in NOG mice

NOD/shi-SCID IL2Rγ^-/-^ (NOG) mice were obtained from Dr. Mamoru Ito (Central Institute for Experimental Animals, Kawasaki, Japan). Mice were bred and maintained under specific pathogen free conditions with acidified water (pH 5.3) at the Animal Core Facility of Gustave Roussy Institute (n° E-94-076-11). Animal experiments were approved by Ethical Committee C2EA-26: Comité d’éthique en expérimentation animale de l’IRCIV ceea26@gmail.com officially registered with the French ministry of research. This project using mice was officially authorized by the French ministry of research (Permit number: 2012–148), as per Directive 2010/63 prescriptions and transposition into French law and regulations.

Mice, 6 to 8 week-old, were irradiated at 2.5 Gy with an IBL637 gamma irradiator containing a Cs-137 source. Mice were intravenously injected 24 hours later with 2x10^6^ EB cells at day 16 in 200 μL PBS or intra-bone marrow transplantation (IBMT) with 3 to 5x10^5^ EB cells at day 16 in 30 μL PBS. Peripheral blood was obtained from the retro-orbital sinus of anesthetized mice and analyzed at 4, 8 and 12 weeks post-transplant for human cells by multicolor flow cytometric analysis as described below. Mice were euthanatized at 12 and 16 weeks post-transplant. Blood, bone marrow (BM) and spleen cells were collected and stained with PE-conjugated mouse anti-human CD45 (BD Biosciences) and analyzed on a FACSCalibur cytometer. When samples were low or negative for human CD45 by FACS analysis, quantitative PCR (Q-PCR) using human specific primers for *CD45* gene was performed to determine the presence of human cells. Genomic DNA was extracted from femurs, tibias, peripheral blood leukocytes and spleen. Human *CD45* forward primer 5’-ACTTTCCCATCTGATCTCTGATTC-3’ and reverse primer 5’-CTGGATTTATCATCTGGGTTTGT-3’ were used to amplify a 201 bp sequence. Mouse actin was used as the housekeeping gene for normalization, forward primer 5’-GTACCACAGGCATTGTGATG-3’ and reverse primer 5’-GCAACATAGCACAGCTTCTC-3’ were used to amplify a 217 bp sequence by Q-PCR according to the manufacturer’s instructions. The Q-PCR was performed and analyzed by using the Light Cycler 480 with SYBR Green Master Mix kit (Roche Diagnostics). The criterion for engraftment was the detection of human DNA.

### Real-time quantitative reverse transcriptase PCR

Total RNA was extracted from cells at pluripotent stage and from EB using RNeasy Mini kit (Qiagen) according to the manufacturer’s instructions. First-strand complementary DNA synthesis was performed using oligo (dT) primers and SuperScript III reverse transcriptase (Fisher Scientific). The resulting complementary DNA was analyzed for differential gene expression by using the Light Cycler 480 with SYBR Green Master Mix kit (Roche Diagnostics). Q-RT-PCR conditions were made according to the manufacturer’s instructions. For each gene a calibration curve was performed with a universal RNA (Clontech), which allows quantifying relative RNA expression of ng RNA/μl. Target gene expression was normalized with the endogenous RNA control human hypoxanthine guanine phosphoribosyl transferase (*HPRT*). The primer sequences of hematopoietic genes: *RUNX1 (*primers which amplified the 3 isoforms a+b+c of *RUNX1*), *HOXB4*, *TAL1*, *PU*.*1*, *GATA1*, *GATA2*, *GATA3*, *MPO and IKAROS*, are shown in Table 2.

**Table 2:** Primer sequences.

Gene	Primers sequences (5′ to 3’)	
	Forward	Reverse
*RUNX1*	TCGGCTGAGCTGAGAAATG	GTGATGGTCAGAGTGAAGCTTTT
*HOXB4*	CTGGATGCGCAAAGTTCAC	CGTGTCAGGTAGCGGTTGTA
*TAL1*	AACGCCAACTGGAGATTTCA	TTCTCGACCAGGATCAAAGC
*PU*.*1*	GTGCAAAATGGAAGGGTTTC	GGAGCTCCGTGAAGTTGTTC
*GATA1*	CCCTGTCCCCAATAGTGCT	CCTGCCCGTTTACTGACAAT
*GATA2*	AAGGCTCGTTCCTGTTCAGA	GGCATTGCACAGGTAGTGG
*GATA3*	GCTTCGGATGCAAGTCCA	GCCCCACAGTTCACACACT
*MPO*	CGTCAACTGCGAGACCAG	GTCATTGGGCGGGATCTT
*IKAROS*	CCTTCCGGGCACACTGTA	TCTCTCTGATCCTATCTTGCACA
*HPRT1*	TAATTGGTGGAGATGATCTCTCAAC	TGCCTGACCAAGGAAAGC

Primer sequences used for Q-PCR of hematopoietic genes detection.

### Cell sorting and *HoxA3* and *RUNX1c* Q-RT-PCR analysis

At day 16 of culture, EB’s were stained with CD34 or its isotype matched IgG-APC conjugated antibody (Beckman Coulter) and sorted for CD34 expression using the FACSDiva instrument (Becton Dickinson). Dead cells were excluded using 7AAD (BD Biosciences) staining.

Total RNA was extracted from CD34+ sorted cells and Q-RT-PCR was performed as described above. The primer sequences of *HoxA3* and *RUNX1c* genes are listed in Table 3. Target gene expression was normalized with the endogenous RNA control human gene excision repair cross-complementation group 3 (*ERCC3*).

**Table 3:** Primer sequences.

Gene	Primers sequences (5′ to 3’)	
	Forward	Reverse
*HoxA3*	CACAAAGCAGAAAACCAGCA	ACAGGTAGCGGTTGAAGTGG
*RUNX1c*	GTGCATTTTCAGGAGGAAGC	TCGTGGACGTCTCTAGAAGGA
*ERCC3*	ACTGGATGGAGCTGCAGAAT	GACATAGGGCACCAGACCTC

### Statistical analysis

All data are expressed as mean ± SEM. Statistical comparisons were performed using a paired Student's t test. Statistical significance was defined as a *p* value <0.05.

## Results

### Myeloid and erythroid differentiation of ES-derived cells

Induction of hematopoiesis from ES cells was assessed using two different methods, EB formation (as described in the materials and methods’ section [16] and [4]) and co-culture with OP9 stromal cell line [8] as described in Fig 1A. Hematopoietic potential was assessed by functional *in vitro* CFC assays and a phenotype of EB-derived cells analyzing the emergence of hematopoietic markers CD34+CD45+, CD34+CD43+ and CD45+CD43+.

In EB at day 16, the number of CFC obtained was different for each ES cell line, SA01 and H1 generated 402±241 CFC (n = 27) and 318±197 (n = 34) respectively, were not considered statistically significant with p values (p>0.05) as determined by unpaired t-test, error bars represent the SEM, whereas H9 and CL01 gave rise to significantly (p>0.05) less CFC than SA01 and H1, 38±12 (n = 3) and 41±27 (n = 3) respectively (Fig 1B). Distribution of hematopoietic colony subtypes (CFU-E, BFU-E, CFU-GM and CFU-GEMM) was similar for SA01 and H1 with a presence of different CFC subtypes unlike H9 and CL01 that exhibited almost exclusive myeloid differentiation potential (Fig 1C). Our results show that, in our culture conditions, the distribution of hematopoietic colony subtypes was different between 4 lines. SA01 and H1 generated more erythroid progenitors (CFU-E and BFU-E) than H9 and CL01, which preferentially gave rise to myeloid cells. Expression of CD34 in EB-derived cells at day 16 varied significantly with 23±9% cells in SA01 (n = 12), 33±16% in H1 (n = 22), 9±2% in H9 (n = 3) and 8±1% in CL01 (n = 3). Differences of expression of CD34 for all cell lines were statistically significant (p<0.05), except for H9 *vs* CL01 (Fig 1D). In addition, we analyzed co-expression of CD34/CD45, CD34/CD43 and CD45/CD43 hematopoietic markers in EB cells from SA01, H1 and H9 cell lines. We found 12±1%, 15±5% and 4±1% CD34+/CD45+; 12±2%, 21±8% and 6±1% CD34+/CD43+ and 8±1%, 9±6% and 2±1% CD45+/CD43+ for SA01, H1 and H9 cell lines (n = 4, 8 and 3 respectively) (Fig 1E and 1F). Our results showed a better expression of hematopoietic markers for ES cell lines with good hematopoietic potential (SA01 and H1) compared with H9 cell line.

Several co-culture studies have used stromal cell lines including OP9 cells to induce hematopoietic differentiation of ES [6,8,17]. We compared the hematopoietic potential of four ES cells after OP9 co-culture. The cells were collected at day 13 and plated in methylcellulose. The number of CFC generated was 139±72 (n = 6) for SA01, 46±22 for H1 (n = 8), 34±12 for H9 (n = 3) and 35±22 for CL01 (n = 3) cell lines. CFC number obtained from the SA01 was significantly different from that obtained from the 3 others ES cell lines (*p*<0.05) (Fig 1G). We observed a similar distribution of hematopoietic colony subtypes for the 4 ES lines to that obtained by the EB method (Fig 1H). The proportion and number of CD34 in ES-derived progenitors obtained on OP9 correlated with the number of CFC generated: 12±7% in SA01 (n = 6), 8±4% in H1 (n = 8), 9±4% in H9 (n = 7) and 6±1% in CL01 (n = 3) were not statistically significant (p>0.05) (Fig 1I). Although several protocols to engage hematopoietic differentiation of PSC exist, we compared the two methods we used (EB and OP9 co-culture) with two other methods described in the literature [4,18] and we found the results were similar for all methods (data not shown).

In conclusion, using two different hematopoietic differentiation protocols, we identified the existence of an intrinsic heterogeneity in the hematopoietic potential of ES regarding the number and commitment of hematopoietic progenitors.

### Myeloid and erythroid differentiation of iPS-derived cells

The hematopoietic differentiation potential was compared between 12 iPS cell lines generated from normal amniotic fluid cells or from homozygous sickle cell anemia (HbS), endothelial progenitor cells from normal peripherical blood and CD34+ from normal cytapheresis or BM. These iPS cell lines were derived by ESTeam Paris Sud (Stem Cell Core Facility, University Paris Sud, France, www.hescreg.eu) using different reprogramming methods. Pluripotency of all iPS cell lines was confirmed by: 1) teratoma assay and histology of representative tissue from three germ layers, 2) expression of pluripotent markers (TRA-1-60, TRA-1-81, SSEA-3 and SSEA-4) by flow cytometry and 3) expression of transgenes, analyzed by reverse transcription polymerase chain reaction (RT-PCR) and iPS cell lines maintaining the expression of transgenes were exclude from this study (Table 1). At the time of induction of hematopoiesis pluripotent markers were checked by flow cytometry (S1A Fig) without any difference between the iPS regarding their hematopoietic potential.

**Fig 2:**
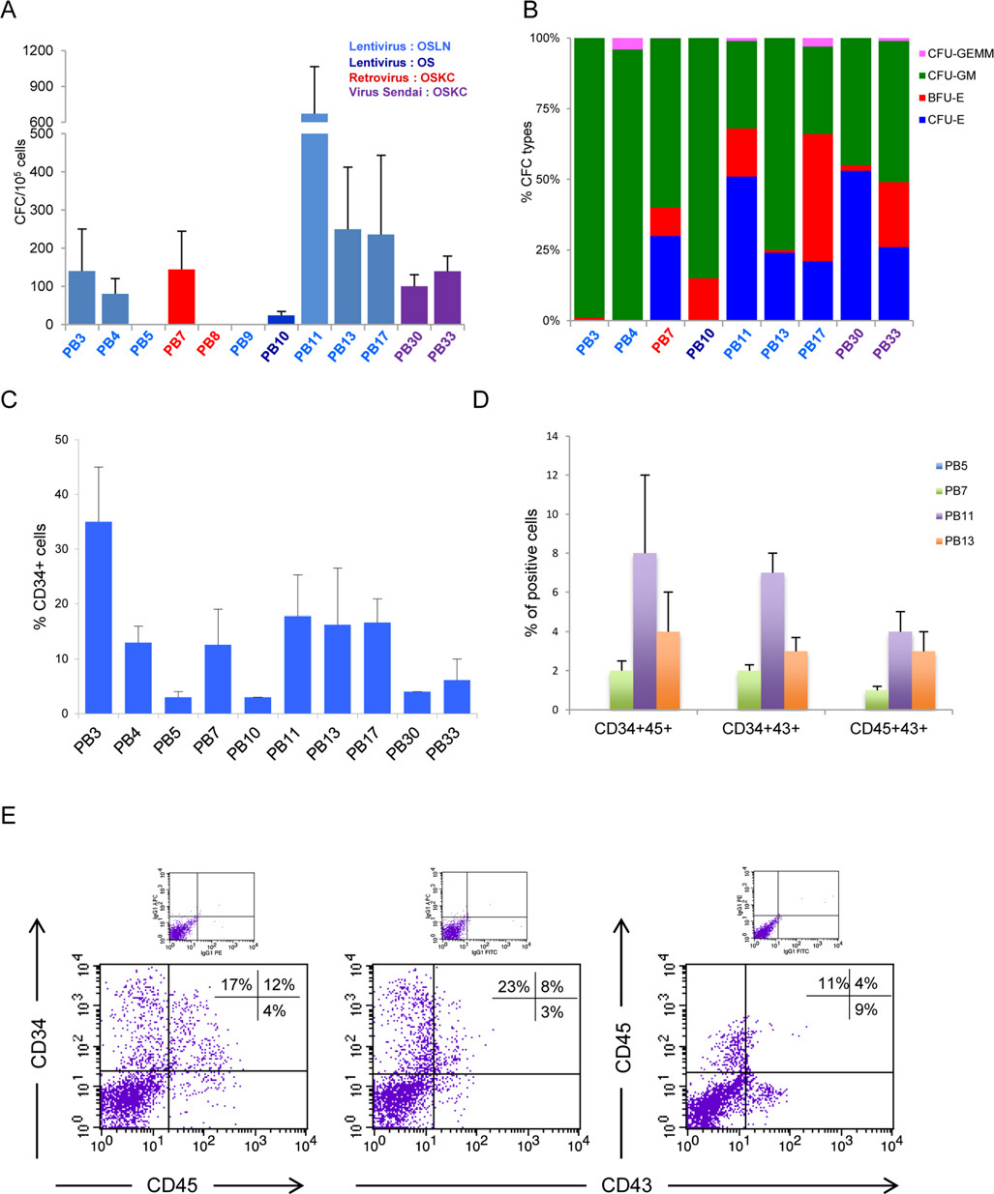
Hematopoietic differentiation potential among several iPS cell lines. Twelve different iPS cell lines were assayed by the EB method. (A) Comparative analysis of total number of CFC. iPS cell lines derived by lentiviral vectors (*OCT4*, *SOX2*, *LIN28* and *NANOG*) in light blue, (*OCT4* and *SOX2*) in dark blue, retroviral vectors (*OCT4*, *SOX2*, *KLF4* and *C-MYC*) in red and by Sendai virus (*OCT4*, *SOX2*, *KLF4 and C-MYC*) in violet, n = 3, 3, 3, 6, 3, 3, 3, 12, 6, 4, 3 and 3, for PB3, PB4, PB5, PB7, PB8, PB9 PB10, PB11, PB13, PB17, PB30 and PB33 cell lines respectively. (B) Types of CFC obtained. Some iPS lines show different colony subtypes, PB7, PB10, PB11, PB13, PB17, PB30 and PB33 cell lines n = 6, 3, 12, 6, 4, 3 and 3, respectively, unlike PB3 (n = 3) and PB4 (n = 3) which differentiate almost exclusively into the myeloid lineage. (C) Flow cytometric analysis of the percentage of CD34+ cells in EB-derived cells, n = 3, 3, 3, 6, 3, 12, 6, 4, 3 and 3, for PB3, PB4, PB5, PB7, PB10, PB11, PB13, PB17, PB30 and PB33 cell lines respectively. (D) EB-derived cells from PB7, PB11 and PB13 co-expressed CD34/CD45, CD34/CD43 and CD45/CD43 hematopoietic markers (n = 3, 3 and 4 respectively), unlike PB5 (n = 3), which do not express these markers. (E) Representative FACS analysis for EB-derived cells from PB11 iPS cell line co-expressed CD34/CD45, CD34/CD43 and CD45/CD43 hematopoietic markers.

Hematopoietic potential was compared among these iPS by using EB model (as described in Fig 1A left) which was, similarly to ES cell lines, heterogeneous. We performed experiments at different passages for each iPS (Table 1) in order to rule out that any hematopoietic variability was due to early *versus* late passages. The CFC numbers varied from 0 to 670 progenitors for 10^5^ EB-derived cells, 140±110 (n = 3), 80±40 (n = 3), 0±0 (n = 3), 144±100 (n = 6), 0±0 (n = 3), 0±0 (n = 3), 24±10 (n = 3), 670±395 (n = 12), 249±163 (n = 6), 235±208 (n = 4), 100±30 (n = 3), 139±40 (n = 3) for PB3, PB4, PB5, PB7, PB8, PB9, PB10, PB11, PB13, PB17, PB30 and PB33 respectively (Fig 2A). Regarding the distribution of CFC subtypes, most iPS (PB7, PB10, PB11, PB13, PB17, PB30 and PB33) exhibited an efficient hematopoietic potential with both erythroid and myeloid lineages and some CFU-GEMM. Two iPS cell lines (PB3 and PB4) were mostly restricted to the myeloid lineage (Fig 2B), whereas three other iPS cell lines (PB5, PB8 and PB9) were unable to generate hematopoietic lineages under our conditions. We found that each iPS had their own intrinsic reproducible behavior in term of number and quality of hematopoietic progenitors. Expression of CD34 in EB-derived cells at day 16 varied significantly, with 35±10% cells in PB3 (n = 3), 13±3% cells in PB4 (n = 3), 3±1% cells in PB5 (n = 3), 13±6% cells in PB7 (n = 6), 3±0.3% cells in PB10 (n = 3), 18±7% cells in PB11 (n = 12), 16±10% cells in PB13 (n = 6), 17±4% cells in PB17 (n = 4), 4±0.2% cells in PB30 (n = 3), 6±4% cells in PB33 (n = 3) (Fig 2C). Subsequently, we analyzed co-expression of CD34/CD45, CD34/CD43 and CD45/CD43 hematopoietic markers in EB cells from iPS (PB5, PB7, PB11 and PB13). Interestingly, PB5 that lacked co-expression of hematopoietic markers did not generate CFC (n = 3). In contrast, for PB7, PB11 and PB13 producing CFC, we found co-expression of CD34/CD45: 2±0.5%, 8±4%, 4±2%; CD34/CD43: 2±0.3%, 7±1%, 3±0.7% and CD45/CD43: 1±0.2%, 4±1%, 3±1% respectively (n = 3, 3 and 4 respectively), but at a lower expression level than in EB’s coming from ES cells (Fig 2D and 2E).

These results indicate that the hematopoietic potential of each iPS was heterogeneous, similarly to ES cell lines. Our data strongly suggests that the variability is not due to lack of pluripotency, the passage number (experiments were performed at various passage numbers for each iPS with similar results), or growth conditions since all cell lines were maintained under the same growth conditions, nor genomic instability since karyotype was normal after iterative passages and concomitant of induction of hematopoiesis (Table 1 and S1A Fig). Our experience shows reproducibility for differentiation into EB's and CFC for both ES and iPS cell lines to differentiate to the hematopoietic lineage *in vitro* (by CFC test). It is noteworthy that in all experiments of CFC hematopoietic differentiation of ES and iPS there is at least one positive control cell line to ensure that the absence of CFCs is not a technical problem (this is also why the N value for some cell lines are larger).

### Reprogramming process did not alter the intrinsic hematopoietic potential of PSC

We investigated whether the reprogramming process would have functional outcomes in the capacity of iPS cell lines to differentiate into hematopoietic myeloid and erythroid lineages. To address this question, we took advantage of the ability to differentiate ES into MSC (non-hematopoietic lineage) and then re-program them to generate isogenic iPS lines that in turn could be directly compared back to their original ES donor state for their hematopoietic potential. For this purpose, we generated isogenic iPS cell lines derived from MSC differentiated from SA01 and H9 (iPS-MSC-SA01 and iPS-MSC-H9) as previously described [14], and then compared them to their pluripotent parental ES cell lines (SA01 and H9) for their hematopoietic potential by EB method [4,19]. At the time of induction of hematopoiesis, pluripotent markers were checked by flow cytometry (S1B Fig) with no apparent differences between parental ES cell lines and iPS-derived from MSC-ES regarding their pluripotency potential.

In order to validate the MSC derived from ES cells have lost their pluripotency capacity and to determine if the reprogramming of these MSC cells (MSC-ES) altered the ES origin cell’s hematopoietic potential, we analyzed expression profile genes involved in hematopoiesis specification in the parental ES cells (SA01 and H9) at the pluripotent stage and compared them to both MSC derived from these cells (MSC-SA01 and MSC-H9) and to iPS-derived from MSC-ES (iPS-MSC-SA01, iPS-MSC-H9). As previously demonstrated, the MSC lost their pluripotent potential [14]. MSC generated from H9 and SA01 were characterized by flow cytometry for markers to MSC differentiation lineage and for negative expression for reprogramming factors *OCT4*, *SOX2*, *NANOG*, *LIN28* and were tested for differentiation along the osteogenic, chondrogenic and adipogenic lineages [14]. To the best of our knowledge no one has shown MSC to be able to differentiate into hematopoietic cells. We then derived iPS-MSC-ES cells (iPS-MSC-SA01 and iPS-MSC-H9) using lentiviral vectors expressing *OCT4*, *SOX2*, *NANOG*, *LIN28* as previously described [14]. The expression profile for different hematopoietic genes (*RUNX1* or *AML1*, *HOXB4*, *TAL1*, *PU*.*1*, *GATA1*, *GATA2*, *GATA3*, *MPO* and *IKAROS)* from MSC-derived from ES cells (MSC-SA01 and MSC-H9) were markedly different compared to their parental ES cells (SA01, H9) and iPS-derived from MSC-ES (iPS-MSC-SA01, iPS-MSC-H9) (S2 Fig).

**Fig 3:**
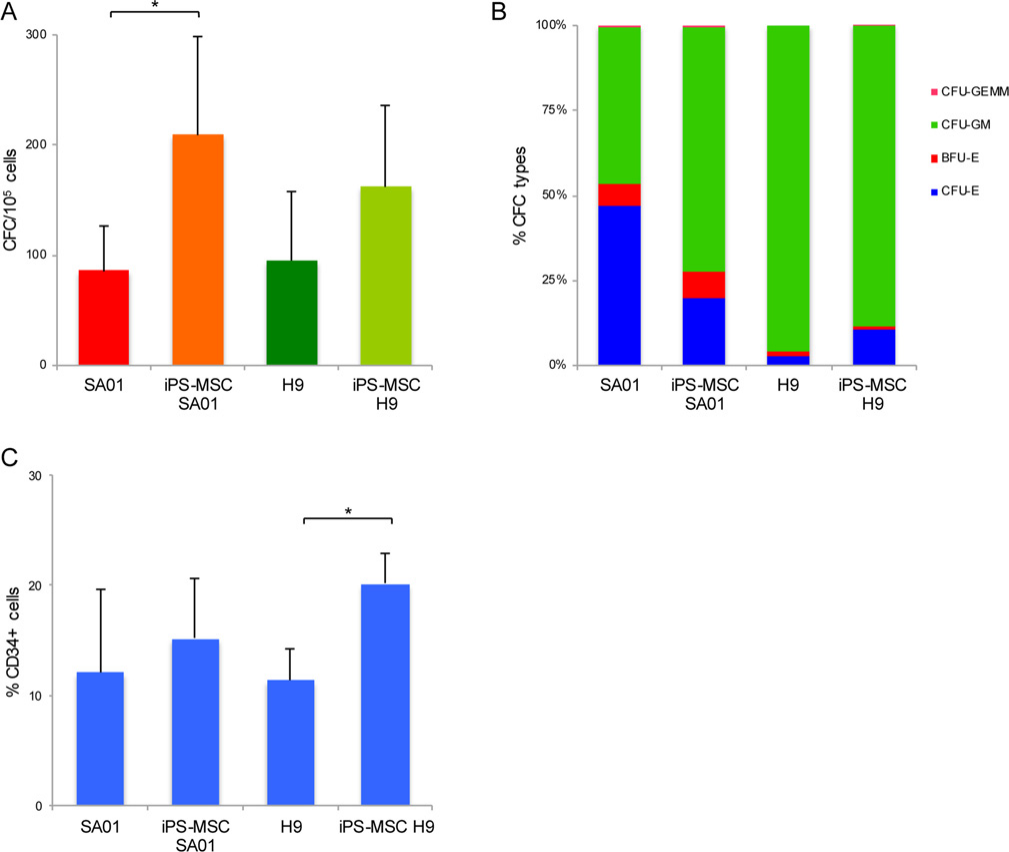
Hematopoietic differentiation potential among different ES cell lines *versus* iPS-MSC-ES. Total number of CFC obtained from SA01 and H9 cell lines and iPS-MSC-SA01 and iPS-MSC-H9 by the EB differentiation method, n = 3 for each assay (A) Number of CFC, data is shown as mean ± s.d. of 4 independent experiments for each cell line, CFC numbers from iPS-MSC-SA01 *vs* parental SA01 cell line was significantly higher (*p<0.05), unlike iPS-MSC-H9 *vs* H9 the difference was not significant (*p*>0.05). (B) Types of CFC from ES and iPS-MSC-ES (n = 4 for each cell line). (C) Flow cytometric analysis of the percentage of CD34+ cells in EB-derived cells from ES and iPS-MSC-ES. Percentage of CD34+ cells for SA01 *vs* iPS-MSC-SA01 were similar (p>0.05) (n = 5), unlike for H9 *vs* iPS-MSC-H9 cell lines percentage of CD34+ cells was statistically significant (*p<0.05) (n = 7).

To determine if the reprogramming of MSC-ES altered its ES origin cell’s ability to generate hematopoiesis, we compared the iPS-MSC-ES to their corresponding ES donor cell line for their hematopoietic potential by CFC assay. The CFC numbers from iPS-MSC-SA01 (n = 4) was significantly higher (*p*<0.05) than parental SA01 (209±90 *vs* 86±40). Similarly, for iPS-MSC-H9, CFC number was higher than parental H9 line but the difference was not significant (162±73 *vs* 95±63, n = 4) (*p*>0.05) (Fig 3A). The subtypes of CFC generated by parental ES and isogenic iPS after reprogramming were similar. Indeed, SA01 and iPS-MSC-SA01 derived-CFC were both erythroid and myeloid with nearly similar proportion of BFU-E, CFU-GM and mixed colonies. The iPS-MSC-H9-derived CFC maintained their mostly myeloid restricted potential and the reprogramming process did not lead to acquired erythroid potential (Fig 3B). EB-derived CD34+ cell proportions at day 16 from SA01 and iPS-MSC-SA01 were similar, 12±7% *vs* 15±5% (n = 5) (p>0.05), unlike for H9 and iPS-MSC-H9 cell lines, we found 11±3% *vs* 20±3% (n = 7) (p<0.05) (Fig 3C) respectively.

For these experiments, the hematopoietic differentiation protocol was different from that used for the EB differentiation to generate data for Figs 1 and 2 as described in the materials and methods’ section [4,19]. In these experiments the FBS and growth factors were changed which might explain the subtle differences observed in the expression level of CD34 and CFC number for SA01 cell line, although it is important to note that the subtypes of CFC remain similar to SA01 and H9 as those shown in Fig 1C.

In conclusion, despite some differences in CFC number and proportion of CD34+ cells, the reprogramming process does not modify the qualitative hematopoietic potential. The variability between iPS for intrinsic hematopoietic potential is more attributable to genetic donor differences rather than to the reprogramming process.

### Xenograft hematopoietic engraftment

Our goal was to assess *in vivo*, the long-term hematopoietic reconstitution potential of ES-derived cells in a xenograft model using immunodeficient NOG mice. We compared EB-derived cells at day 16 from SA01 and H9 with their respective isogenic iPS cell line (iPS-MSC-SA01 and iPS-MSC-H9). When EB’s were intravenously injected, we did not observe any hematopoietic engraftment in the transplanted mice while cord blood CD34+ cells engrafted efficiently (data not shown). To circumvent any caveat due to homing defect, we injected 3 to 5x10^5^ day 16 EB cells directly into one femur of sublethally irradiated NOG mice by intra-BM transplantation (IBMT). Mice were monitored for human reconstitution by peripheral blood analysis at various time points (4, 8, 12–16 weeks) after transplant. Mice were bled and analyzed for human CD45 expression by FACS analysis and Q-PCR assay (data not shown).

The specificity of human CD45 antibody for human cells was confirmed by comparing anti-mouse CD45 *versus* anti-human CD45 staining using non-transplanted control mice (S3A Fig). In addition, we confirmed the specific detection of human cells in EB’s from H9 cell line (S3B Fig top and lower right) and ruled out non-specific binding of anti-CD45 antibody by comparing anti-mouse CD45 (S3B Fig lower middle) and anti-human CD45 staining to their respective isotype control antibodies (S3B Fig lower left).

**Fig 4:**
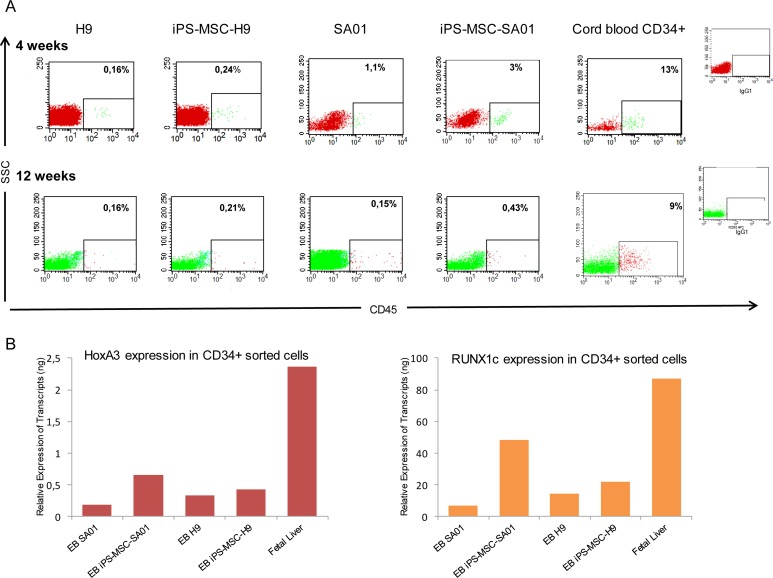
Human engraftment of NOG mice transplanted with ES or iPS cell lines. EB cells were injected directly into the femur of non-lethally irradiated NOG mice. (A) Representative FACS analysis for detection of human CD45+ cells in the blood and BM of transplanted mice. Percentage of human CD45+ cells in blood at 4 weeks (top) and in injected femur at 12 weeks post-transplant (bottom): H9, iPS-MSC-H9, SA01, iPS-MSC-SA01 and control mouse transplanted with human cord blood CD34+ cells and isotype control. (B) Quantitative RT-PCR analysis for *HoxA3* and *RUNX1c* expression in CD34+ EB derived cells from ES and iPS-MSC-ES cell lines, compared to CD34+ human fetal liver (n = 2).

Four weeks after xenograft by EB-derived cells from H9, iPS-MSC-H9, SA01 and iPS-MSC-SA01, we detected a significant percentage of human CD45+ cells (range of 0.02% to 7%) in blood of all mice analyzed by FACS (Table 4) as shown by representative FACS analysis (Fig 4A top). However, the levels of human CD45 positive cells were lower than those found in mice transplanted with human cord blood CD34+ cells (range of 4% to 13% human CD45+ cells). Between 12 to 16 weeks post transplantation, mice were sacrificed and peripheral blood and BM from each injected and un-injected femurs were analyzed separately. While the level of engraftment by cord blood CD34+ cells when injected directly into the femur of mice was lower than that observed when injected using intravenous route (data not shown), engraftment was clearly detected with 5% to 9% of human CD45+ cells in the injected femur and 1% to 4% in the contralateral femur. We also found detectable H9-derived graft EB cells in 6/13 mice with a range of 0.1% to 0.4% human CD45+ cells and iPS-MSC-H9-derived graft EB cells in 5/10 mice with a range of 0.16% to 5% human CD45+ cells (Table 4) and as shown by representative FACS analysis (Fig 4A bottom). Human engraftment was detected in the total BM and injected femur in all transplanted mice with SA01-derived EB (8/8 mice) with a range of 0.01% to 0.5% human CD45+ cells and iPS-MSC-SA01-derived EB (12/12 mice) with a slightly higher percentage of human CD45+ cells (0.01% to 5%). The level of human CD45 positive cells seems slightly higher in mice transplanted with EB-derived cells from iPS-MSC-ES cells than in mice transplanted with EB-derived cells from ES cell lines in agreement with the CFC number, but remains lower than in control mice transplanted with human cord blood CD34+ cells (Table 4). Spleen and thymus were also analyzed. No developed lymph nodes or tumors were observed in any of the examined mice.

**Table 4:** Human engraftment of NOG mice transplanted with ES or iPS cells.

	Blood	Left femur	Right femur	Mice
	(4 w)	(12–16 w)	(12–16 w)	number
H9	0.05–7%	0.1–0.4%	0.1–0.4%	13
iPS-MSC-H9	0.02–5%	0.16–5%	0.2–0.9%	10
SA01	5–6%	0.01–0.5%	0.01–0.2%	8
iPS-MSC-SA01	4–6%	0.01–5%	0.1–0.9%	12
Human CB	4–13%	5–9%	1–4%	7

EB cells were injected directly into the femur of non-lethally irradiated NOG mice. Percentage of human CD45+ cells in blood at 4 weeks and in BM at 12–16 weeks post-transplant: H9, iPS-MSC-H9, SA01, iPS-MSC-SA01 and control mice transplanted with human cord blood (CB) CD34+ cells.

In order to identify potential factors that may prevent ES and iPS derived CD34+ cells from reconstituting immune compromised mice, we analyzed *HoxA3* and *RUNX1c* gene expression from isolated EB’s CD34+ cells from SA01, H9, iPS-MSC-SA01 and iPS-MSC-H9 cell lines and compared them to human fetal liver CD34+ cells (provided by Dr E. Oberlin) which have a high capacity to reconstitute immunodeficient mice. We found that EB’s CD34+ populations from both ES and iPS-MSC-ES have a much lower expression for *HoxA3* and *RUNX1c* genes than fetal liver CD34+ cells (Fig 4B).

In conclusion, a weak intrinsic hematopoietic reconstitution activity seems to also be observed among ES and iPS cell lines, but considering the low level of hematopoietic reconstitution by cells generated from ES and iPS-MSC-ES compared to human cord blood CD34+ cells, we believe that these cells are distinct from adult hematopoietic reconstituting cells and possess limited ability to proliferate, nest and perhaps migrate *in vivo*. Regardless, robust engraftment and hematopoietic reconstitution of immune compromised mice from ES and iPS remains an elusive issue that has yet to be resolved.

### Expression of transcription factors does not distinguish PSC with good *versus* poor hematopoietic output

**Fig 5:**
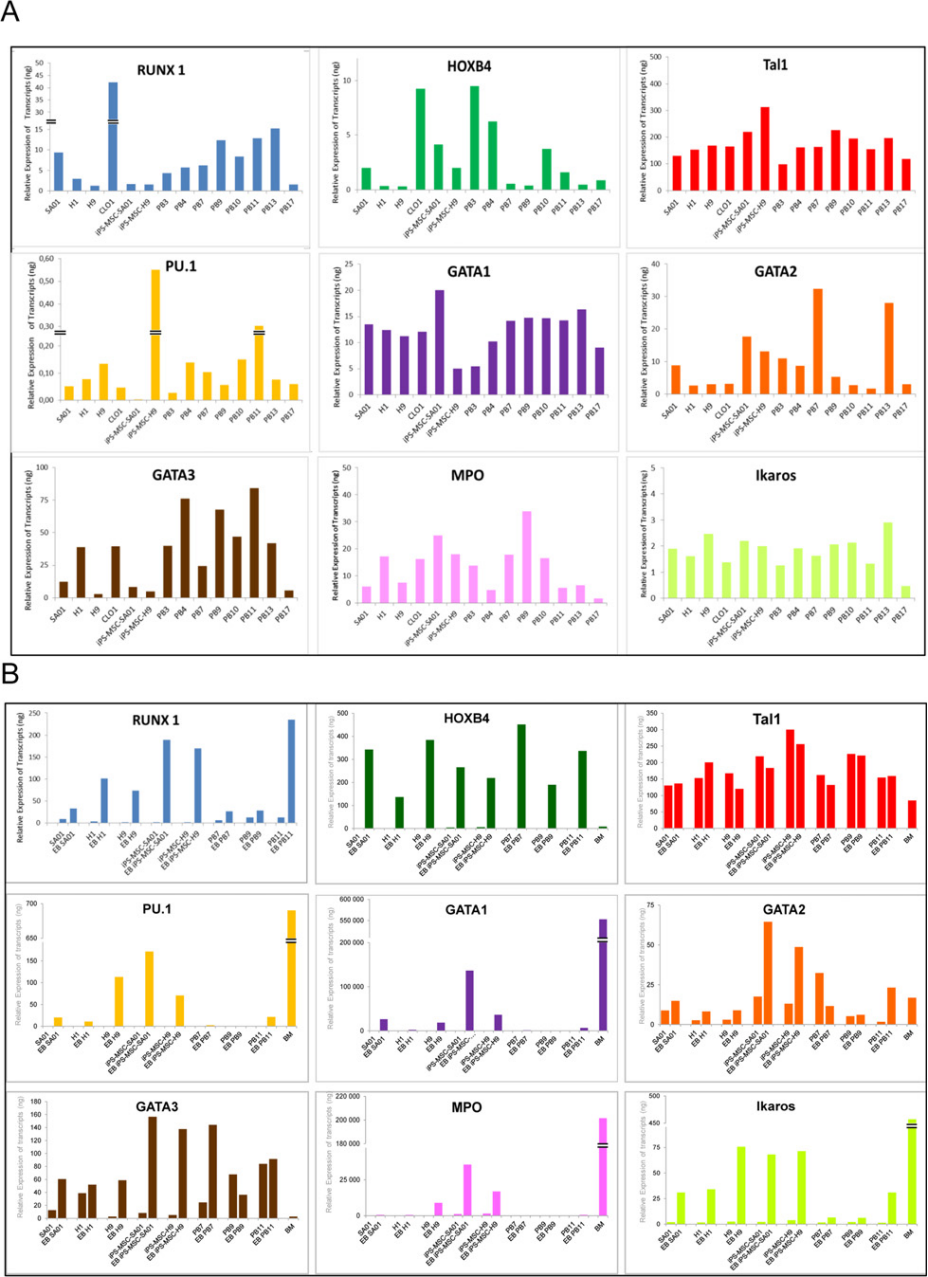
Expression of hematopoietic transcription factors for ES and iPS cell lines. Gene expression level of *RUNX1*, *HOXB4*, *TAL1*, *PU*.*1*, *GATA1*, *GATA2*, *GATA3*, *MPO* and *IKAROS* was evaluated by Q-RT-PCR (A) at pluripotent stage and (B) after differentiation in EB at day 16 and human BM as a positive control of expression hematopoietic genes. Gene expression was normalized relative to the endogenous RNA control human *HPRT*.

We investigated whether the variation in hematopoietic differentiation potential among different ES and iPS cell lines could be predicted by expression of key genes involved in hematopoiesis at pluripotent stage and after differentiation in EB at day 16 by Q-RT-PCR. First it was important to look at the pluripotent stage to determine if any of the cell lines that had good hematopoiesis expressed hematopoietic genes that might give them an advantage for hematopoiesis over the poor hematopoietic lines which could them be used for screening new iPS cell lines. Expression profile of specific genes involved in early hematopoietic commitment and in myelo-lymphoid specification, *RUNX1 (*isoforms a+b+c), *HOXB4*, *TAL1*, *PU*.*1*, *GATA1*, *GATA2*, *GATA3*, *MPO* and *IKAROS* was studied at the pluripotent stage for four ES cell lines (SA01, H1, H9 and CL01), and 10 iPS cell lines from different origin (iPS-MSC-SA01, iPS-MSC-H9, PB3, PB4, PB7, PB9, PB10, PB11, PB13 and PB17) (Fig 5A). Relative expression of 3 isoforms to *RUNX1* was similar for all cell lines except for CL01 which has a poor hematopoietic potential. Similar *HOXB4* level expression was observed for all cell lines. *TAL1*, also called *SCL* had almost the same relative expression for all cell lines with a slightly more elevated level for iPS-MSC-H9 cell line. *PU*.*1/SPI1* had a relatively low expression when compared to the previous transcription factors except for iPS-MSC-H9 and PB11, which had a 3 to 6 fold higher expression. *GATA1* expression was similar for all cell lines except for iPS-MSC-H9 and PB3 where it was lower than for the others cell lines; interestingly, these two cell lines have a low differentiation potential toward the erythroid lineage. The *GATA2* transcription factor’s relative expression was elevated in PB7 and PB13 cell lines that demonstrated a robust hematopoietic potential. *GATA3* expression showed significant differences in its expression at the pluripotent stage between different cell lines. Myeloperoxidase (*MPO*) varied according to cell lines and virtually had very little expression in PB17 cell line. Finally, *IKAROS* had a moderate expression for all lines with collapsed in PB17 (Fig 5A). In conclusion, a large variation in the level of gene expression at the pluripotent stage was observed but was not able to be directly correlated to distinguish PSC lines with greater hematopoietic potential. These results suggest that intrinsic hematopoietic gene expression at the pluripotent stage probably is not the contributing factor for their hematopoiesis potential but rather that the cell’s origin and external signals play a dominant role in determining their hematopoietic differentiation potential.

Thereafter, we examined the expression of these genes during differentiation (day 16 EBs) in comparison to the pluripotent stage for three ES cell lines (SA01, H1 and H9), two iPS-MSC-ES cell lines (iPS-MSC-SA01 and iPS-MSC-H9) and three iPS cell lines (PB7, PB9 and PB11) and human BM as a positive control for hematopoietic gene expression. Gene expression was normalized relative to the endogenous RNA control human *HPRT*. All mRNA expression was increased at the EB stage of development compared to the pluripotent stage with the exception of *TAL1*, which expression was similar at both developmental time points for all cell lines. We also detected very high levels of expression for *GATA1* mRNA for iPS-MSC-SA01 EBs compared to the pluripotent state. As expected, the expression level of these key hematopoietic factors in EB varied during hematopoietic differentiation (Fig 5B).

## Discussion

In this study, we found a significant intrinsic variation among human ES and iPS cell lines in hematopoietic differentiation, regarding CFC potential both quantitatively and qualitatively and in hematopoietic *in vivo* engraftment and reconstitution. It is known that not all PSC have the same capacity to differentiate into desired cell types. A previous study using ES cells showed that erythroid differentiation did not occur or albeit at low levels, depending on the cell line studied [20]. Like other investigators [12], we have demonstrated that some ES and iPS cell lines have a better capacity to differentiate *in vitro* into hematopoietic lineage than other cell lines with quantitative and qualitative differences, in particular a remarkable difference in the ratio of myeloid *versus* erythroid CFC. It has been recently reported that iPS cells differ and retained residual DNA methylation patterns typical of parental somatic cells [21]. Nevertheless, the influence of epigenetic memory from somatic origin cell type in differentiation capacities of iPS cells remains controversial. It is known that PSC lines varied in differentiation efficiency and reproducibly but these differences were not correlated to the source of the cells [22] in addition, it was reported hematopoietic differentiation variation in five human ES cell lines and variation in hematopoietic and osteoclast differentiation in three human ES cell lines [10,23]. A similar hematopoietic induction potential and erythroid differentiation pattern was reported among iPS lines from human neural or cord blood cells [24]. Other studies showed that epigenetic memory could disturb the ability of iPS to differentiate into cell types that are different from their cells of origin. Daley’s group showed that iPS can retain a residual epigenetic memory of the original cell type and accordingly the donor cell type can influence the epigenome and differentiation potential of iPS cells and genetic and epigenetic variations contribute to functional variability between cell lines [22,25]. However, continuous long-term passaging of iPS seems to abrogate transcriptional, epigenetic and functional differences [26]. Our study confirmed that donor cell origin is one important determinant of variation in hematopoietic potential of iPS and ES cell lines, rather than the reprogramming process. A few iPS cell lines (PB3, PB4, PB11, PB13 and PB17) derived with lentiviruses appear to have good hematopoietic potential but this does not allow us to conclude that this method is better since other iPS cell lines (PB5 and PB9) derived with the same lentiviruses had no hematopoietic differentiation. Furthermore, iPS-derived MSC-ES cells from SA01 and H9 ES cell lines reproduce the similar hematopoietic potential in term of myeloid and erythroid lineages compared to the original donor parental SA01 and H9 ES cell lines. This data strongly suggests that the donor cell of origin rather than the re-programming method plays the dominant role in iPS hematopoiesis differentiation, which has also been proposed as a possible mechanism by other groups in the iPS field. A recent study by Yamanaka’s group showed that the methods used to derive iPS does not influence the hepatic potential and that hepatic differentiation efficiency was linked to the genetic background of the donors [27]. The comparison of hepatic differentiation ability from peripheral blood-derived iPS or dermal fibroblasts-derived iPS of a same donor was similar, confirming that variability leads to the genetic diversity of the donor and not the type of somatic cells (peripheral blood or dermal fibroblasts).

To date, PSC-derived cells have demonstrated only limited potential for long-term multilineage hematopoietic engraftment *in vivo* [28–30]. Generation of transplantable hematopoietc stem cells from ES and iPS lines was very inefficient and variable from different PSC. We identified differences in expression patterns of *HoxA3* and *RUNX1c* between EB’s CD34+ from 2 ES and 2 iPS-MSC-ES cell lines compared to human fetal liver CD34+ cells. Kaufman’s group recently demonstrated deficient expression from the *HoxA* locus in derived progenitors CD34+43+ cells from ES and iPS cell lines compared to CD34+ cells from human umbilical cord blood [31]. Our results are in agreement with their work suggesting that deficient *RUNX1* and *HoxA* family gene expression may contribute to the inability of human ES and iPS-derived hematopoietic cells to reconstitute immune deficient mice. Other authors have shown that definitive hematopoiesis is regulated by *HoxA* cluster genes expressed mainly in hematopoietic stem cells suggesting that *HoxA* cluster expression is important for adult hematopoiesis in mice [32]. Ramos-Mejia and colleagues show that *HoxA9* plays a major role in hematopoiesis and leukemogenesis but *HoxA9* alone is not sufficient to confer long-term engraftment potential *in vivo* for human ES-hematopoietic derivatives [33]. Kiba’s group showed that *HoxA3* has a key role in the commitment to hematopoiesis *versus* endothelial differentiation in mice, restrained hematopoietic differentiation of the earliest endothelial progenitors and *RUNX1* have a essential role to extinguish an endothelial program [34]. Challen and colleagues showed increased *Runx1c* expression in the AGM region in mouse embryos and they also showed that in mouse ES cells *Runx1c* expression to be specific for emerging definitive hematopoiesis, unlike *Runx1a* and *Runx1b* which are expressed throughout hematopoiesis [35]. Our results are consistent with the recent work of Kaufman’s group, we showed that the CD34+ population from EB's derived from ES and iPS express considerably less of the transcripts for *HoxA3* and *RUNX1c* compared to CD34+ cells that have a high capacity for reconstituting immune deficient mice.

The inability to reconstitute long-term hematopoiesis could be explained by the fact that they might contain only embryonic primitive hematopoietic progenitors from corresponding yolk sac [8,19,36], which requires Wnt-β catenin signaling to differentiate to definitive hematopoietic progenitors [37] or over-expression of *RUNX1* [38]. Failure to regenerate multilineage hematopoiesis *in vivo* was also attributed to the inability to down-regulate key microRNAs involved in hematopoiesis [39] or a lack of providing specific environmental stromal cell-derived factors. Ledran and colleagues could obtain long-term engraftment in immunodeficient recipients with the ability to engraft secondary transplant mice from ES-derived cells co-cultured on stromal cell lines [28]. Recent studies reported that teratoma formation could favor the emergence of engraftable hematopoietic stem cells from iPS lines [40,41]. Recently, Gori and colleagues, showed that endothelial Notch ligands promote PSC-definitive hematopoiesis and production of long-term engrafting cells [42].

We compared the ES and iPS cell lines at the pluripotent stage for the expression of intrinsic hematopoietic factors that could be used as a predictor for determining their hematopoiesis potential. Expression of either early or late hematopoietic transcription factors at the pluripotent stage in ES and iPS could not predict the blood developmental potential *in vitro*. In the four ES and 10 iPS cell lines we examined, none of the analyzed genes demonstrated a specific correlation for determining between good *versus* poor hematopoietic potential. These findings support the notion that the level of expression for genes involved in early and late stages of hematopoietic differentiation is not a feature of prediction for blood developmental potential of ES and iPS cell lines to differentiate *in vitro*. Our results are in agreement with the study of Ramos-Mejia [43] showing differences in expression level of hematopoietic transcription factors between good *versus* poor hematopoietic iPS lines. These results suggests there is an unpredictable variation in hematopoietic differentiation for ES and iPS cell lines that can not be predicted based on phenotype or gene expression of the undifferentiated pluripotent cells lines.

In summary, our results demonstrate the capacity of ES and iPS to differentiate into hematopoietic cells *in vitro*. Nevertheless, there exist some quantitative and qualitative differences about hematopoietic differentiation between the ES and iPS cell lines used, in particular there were remarkable differences in the ratio of myeloid *versus* erythroid CFC. Our results suggest that the genetic background of the original cells has a major influence on the iPS cell lines’ hematopoietic differentiation. This means that the cells’ origin, rather than the re-programming method, passage number or method of differentiation is probably the major determinant of variation in hematopoietic differentiation.

## Supporting Information

S1 Fig Expression of pluripotent markers in ES and iPS cell lines by flow cytometry. All ES and iPS cell lines used in this work were routinely screened for high levels of stem cell pluripotency gene expression. Representative FACS analysis of TRA-1-60, TRA-1-81, SSEA-3 and SSEA-4 expression for (A) H1, PB7, PB11 and PB13 cell lines (at passage 48, 48, 41 and 30 respectively). (B) SA01, iPS-MSC-SA01, H9 and iPS-MSC-H9 cell lines (at passage 42, 49, 48 and 58 respectively).
(TIF)

S2 Fig Expression of hematopoietic transcription factors for ES *versus* MSC *versus* iPS-MSC-ES cell lines. Relative gene expression level of *RUNX1*, *HOXB4*, *TAL1*, *PU*.*1*, *GATA1*, *GATA2*, *GATA3*, *MPO* and *IKAROS* was determined by Q-RT-PCR at the pluripotent stage in the parental ES cells (SA01 and H9) and then compared them to both MSC derived from these cells (MSC-SA01 and MSC-H9) and to iPS-derived from MSC-ES (iPS-MSC-SA01, iPS-MSC-H9).
(TIF)

S3 Fig Human engraftment of NOG mice transplanted with ES or iPS cell lines. EB cells were injected directly into the femur of non-lethally irradiated NOG mice. (A) Representative FACS analysis for non-transplanted control mouse blood, showing specificity of mouse CD45 (middle) *versus* human CD45 (right) with Ig-isotype controls (left). The mouse was a control for the transplanted experimental group and bled at the 4 weeks experimental time points. Note the human CD45 antibody is extremely specific and no human cells or non-specific background was detected compared to mouse CD45 and isotype controls. (B) Representative FACS analysis for mouse blood at 4 weeks post-transplant with EB’s from H9 cell line double stained for mouse-CD45 and human-CD45 antibody. Note the specificity of the human-CD45 to detect a small but distinct cell population as shown in the bottom right dot plot.
(TIF)
