# Supplementary figures and images for: Donor Dependent Variations in Hematopoietic Differentiation among Embryonic and Induced Pluripotent Stem Cell Lines
Source: PLoS One. 2016 Mar 3;11(3):e0149291. doi: 10.1371/journal.pone.0149291 (PMC4777368; doi:10.1371/journal.pone.0149291)

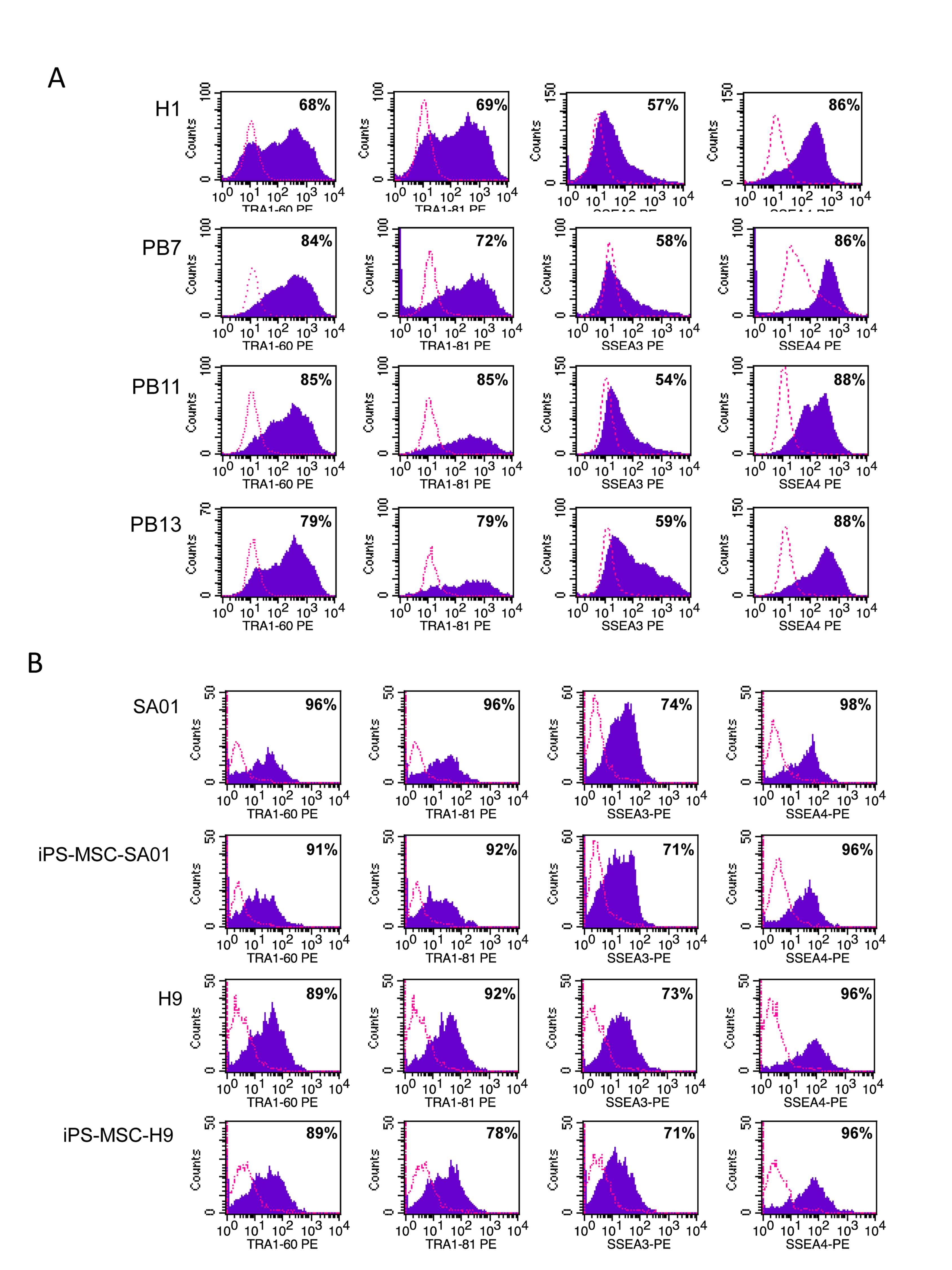

Supplement: S1 Fig — All ES and iPS cell lines used in this work were routinely screened for high levels of stem cell pluripotency gene expression. Representative FACS analysis of TRA-1-60, TRA-1-81, SSEA-3 and SSEA-4 expression for (A) H1, PB7, PB11 and PB13 cell lines (at passage 48, 48, 41 and 30 respectively). (B) SA01, iPS-MSC-SA01, H9 and iPS-MSC-H9 cell lines (at passage 42, 49, 48 and 58 respectively). (TIF) [file pone.0149291.s001.tif]

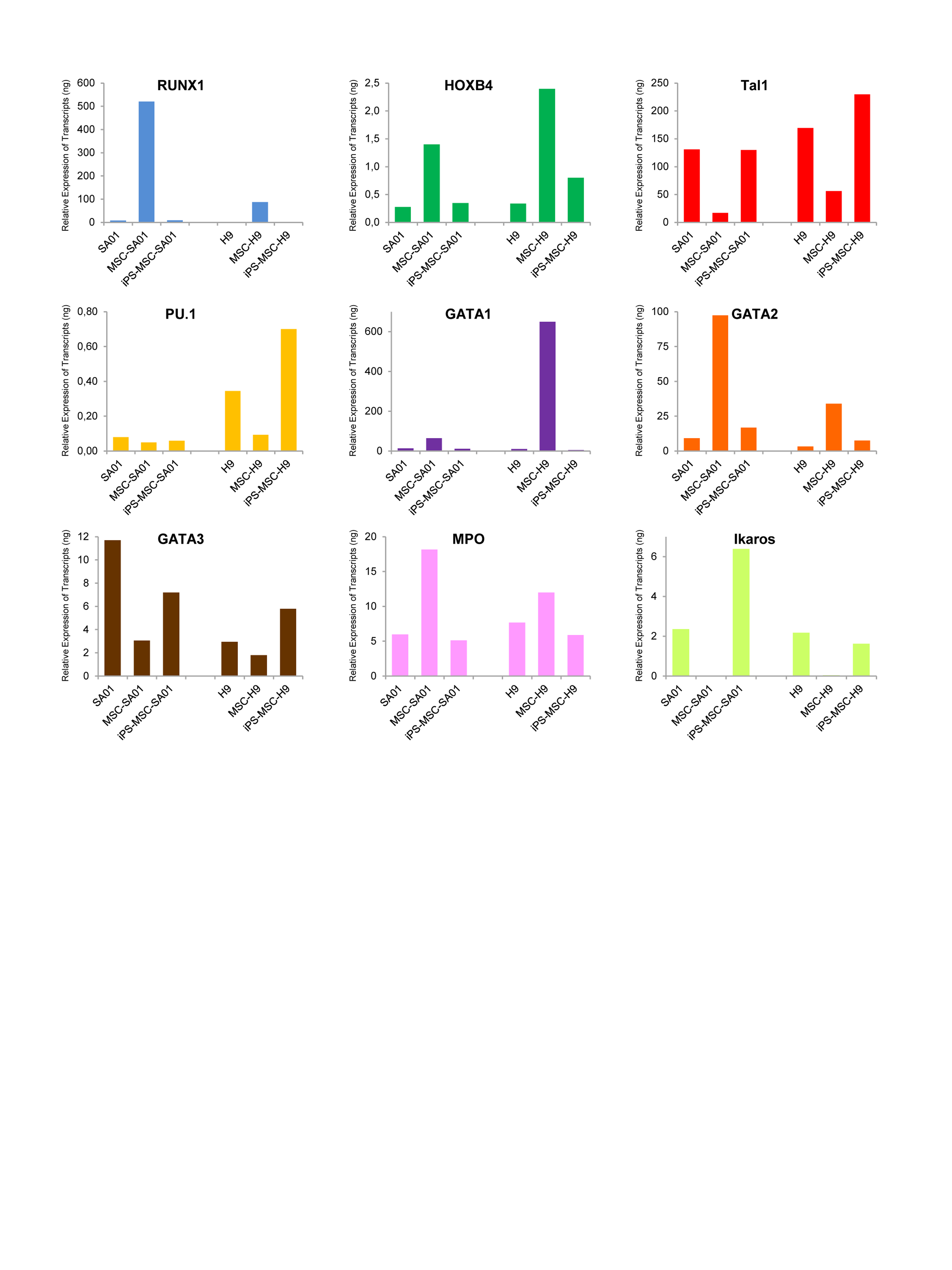

Supplement: S2 Fig — Relative gene expression level of RUNX1, HOXB4, TAL1, PU.1, GATA1, GATA2, GATA3, MPO and IKAROS was determined by Q-RT-PCR at the pluripotent stage in the parental ES cells (SA01 and H9) and then compared them to both MSC derived from these cells (MSC-SA01 and MSC-H9) and to iPS-derived from MSC-ES (iPS-MSC-SA01, iPS-MSC-H9). (TIF) [file pone.0149291.s002.tif]

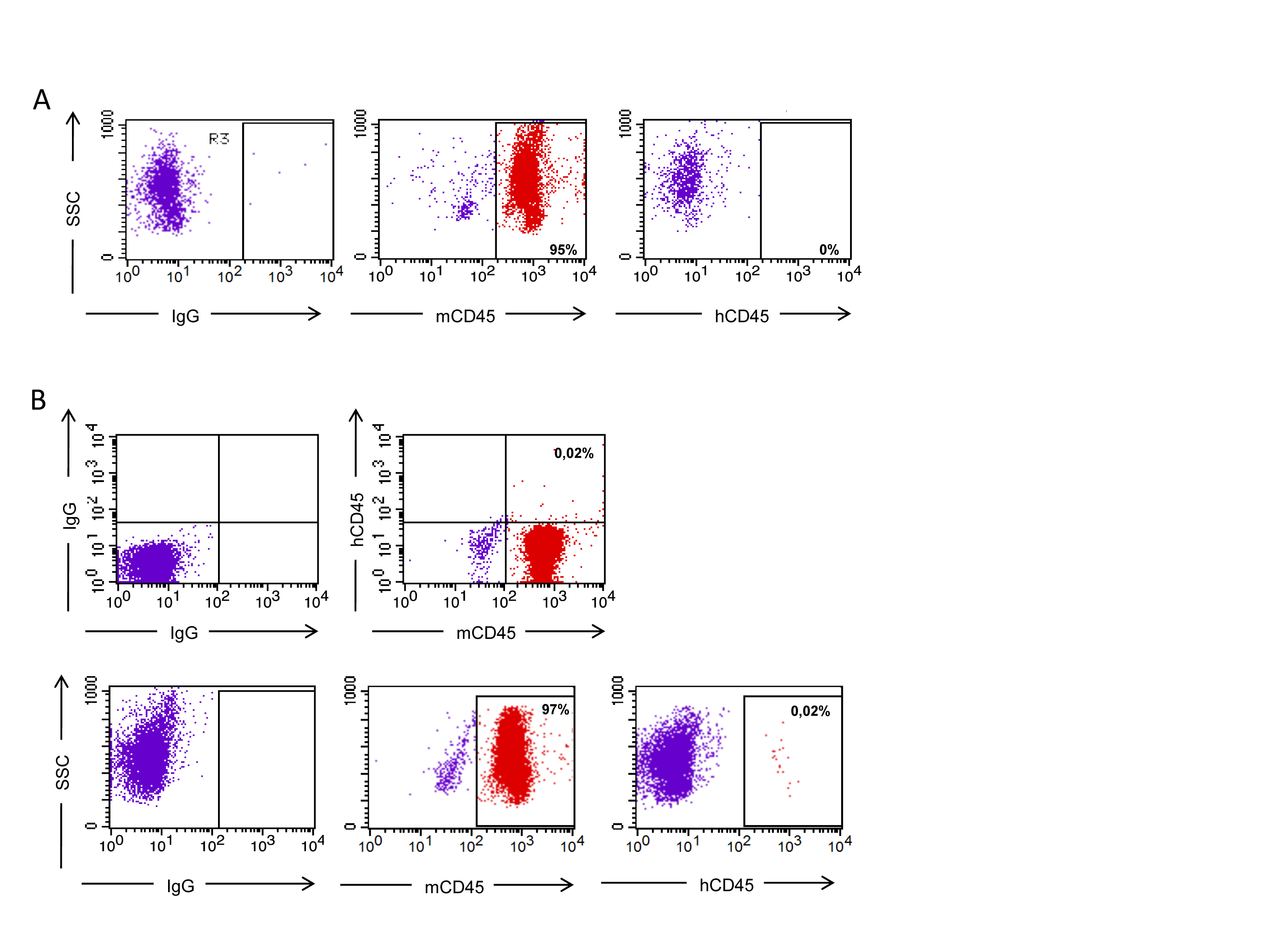

Supplement: S3 Fig — EB cells were injected directly into the femur of non-lethally irradiated NOG mice. (A) Representative FACS analysis for non-transplanted control mouse blood, showing specificity of mouse CD45 (middle) versus human CD45 (right) with Ig-isotype controls (left). The mouse was a control for the transplanted experimental group and bled at the 4 weeks experimental time points. Note the human CD45 antibody is extremely specific and no human cells or non-specific background was detected compared to mouse CD45 and isotype controls. (B) Representative FACS analysis for mouse blood at 4 weeks post-transplant with EB’s from H9 cell line double stained for mouse-CD45 and human-CD45 antibody. Note the specificity of the human-CD45 to detect a small but distinct cell population as shown in the bottom right dot plot. (TIF) [file pone.0149291.s003.tif]
